# *N*-(Pyrazin-2-yl)aniline

**DOI:** 10.1107/S1600536808031942

**Published:** 2008-10-11

**Authors:** Wan Ainna Mardhiah Wan Saffiee, Azila Idris, Zanariah Abdullah, Zaharah Aiyub, Seik Weng Ng

**Affiliations:** a Department of Chemistry, University of Malaya, 50603 Kuala Lumpur, Malaysia

## Abstract

The two aromatic rings in the title compound, C_10_H_9_N_3_, are inclined at 15.2 (1)° to each other; this opens up the angle at the amino N atom to 130.4 (1)°. The amino N atom forms a hydrogen bond to the 4-N atom of an adjacent mol­ecule to create a chain motif.

## Related literature

For the structure of amino­pyrazine, see: Chao *et al.* (1976 ▶). For the structure of 2-pyrazinyl-*N*-2-nitro­phenyl­aniline; see: Parsons *et al.* (2006 ▶).
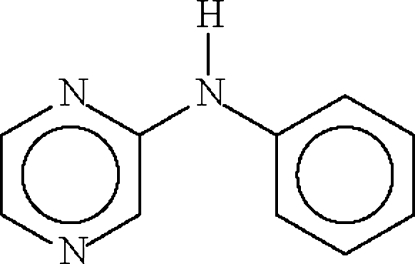


## Experimental

### 

#### Crystal data

C_10_H_9_N_3_
*M* *_r_* = 171.20
Monoclinic, 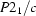

*a* = 11.0644 (3) Å
*b* = 7.8423 (3) Å
*c* = 10.8907 (3) Å
β = 116.439 (2)°
*V* = 846.15 (5) Å^3^
*Z* = 4
Mo *K*α radiation
μ = 0.09 mm^−1^
*T* = 100 (2) K
0.20 × 0.10 × 0.05 mm

#### Data collection

Bruker SMART APEX diffractometer
Absorption correction: none
5664 measured reflections
1934 independent reflections
1463 reflections with *I* > 2σ(*I*)
*R*_int_ = 0.033

#### Refinement

*R*[*F* ^2^ > 2σ(*F* ^2^)] = 0.041
*wR*(*F* ^2^) = 0.101
*S* = 1.03
1934 reflections
122 parameters
1 restraint
H atoms treated by a mixture of independent and constrained refinement
Δρ_max_ = 0.23 e Å^−3^
Δρ_min_ = −0.23 e Å^−3^

### 

Data collection: *APEX2* (Bruker, 2007 ▶); cell refinement: *SAINT* (Bruker, 2007 ▶); data reduction: *SAINT*; program(s) used to solve structure: *SHELXS97* (Sheldrick, 2008 ▶); program(s) used to refine structure: *SHELXL97* (Sheldrick, 2008 ▶); molecular graphics: *X-SEED* (Barbour, 2001 ▶); software used to prepare material for publication: *publCIF* (Westrip, 2008 ▶).

## Supplementary Material

Crystal structure: contains datablocks global, I. DOI: 10.1107/S1600536808031942/pk2121sup1.cif

Structure factors: contains datablocks I. DOI: 10.1107/S1600536808031942/pk2121Isup2.hkl

Additional supplementary materials:  crystallographic information; 3D view; checkCIF report

## Figures and Tables

**Table 1:** Hydrogen-bond geometry (Å, °)

*D*—H⋯*A*	*D*—H	H⋯*A*	*D*⋯*A*	*D*—H⋯*A*
N1—H1⋯N2^i^	0.89 (1)	2.12 (1)	2.977 (2)	162 (1)

Symmetry code: (i) 
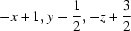
.

**Fig. 1.**
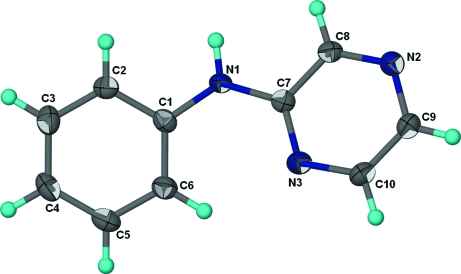
Thermal ellipsoid plot (Barbour, 2001) of C10H9N3 at the 70% probability level; hydrogen atoms are drawn as spheres of arbitrary radius.

## supplementary crystallographic information

### Comment

There are few structural examples of pyrazine compounds having an amino substituent; these are limited to, for example, aminopyrazine (Chao *et al.*, 1976) and pyrazinyl-*N*-2-nitrophenylaniline (Parsons *et al.*, 2006). In the title compound (Scheme I, Fig. 1), the two aromatic rings are aligned at 15.2 (1)°; these open up the angle at the amino nitrogen to 130.4 (1) °. The amino nitrogen forms a hydrogen bond to the 4-nitrogen atom of an adjacent molecule to furnish a chain motif.

### Experimental

Chloropyrazine (1 ml, 1.1 mmol) and aniline (1 ml, 1.1 mmol) were heated at 423–433 K for 3 h. The solid was dissolved in water. The compound was extracted with ether. The ether extract was dried over sodium sulfate; evaporation of the solvent gave a colorless crystals among some unidentified dark brown materials.

### Refinement

Carbon-bound H-atoms were placed in calculated positions (C—H 0.95 Å) and were included in the refinement in the riding model approximation, with *U*(H) fixed at 1.2*U*(C). The amino H-atom was located in a difference Fourier map, and was refined with a distance restraint of N—H 0.88 (1) Å.

### Crystal data

**Table:**

C_10_H_9_N_3_	*F*(000) = 360
*M**_r_* = 171.20	*D*_x_ = 1.344 Mg m^−^^3^
Monoclinic, *P*2_1_/*c*	Mo *K*α radiation, λ = 0.71073 Å
Hall symbol: -P 2ybc	Cell parameters from 3723 reflections
*a* = 11.0644 (3) Å	θ = 3.3–26.4°
*b* = 7.8423 (3) Å	µ = 0.09 mm^−^^1^
*c* = 10.8907 (3) Å	*T* = 100 K
β = 116.439 (2)°	Prism, colourless
*V* = 846.15 (5) Å^3^	0.20 × 0.10 × 0.05 mm
*Z* = 4	

### Data collection

**Table:**

Bruker SMART APEX diffractometer	1463 reflections with *I* > 2σ(*I*)
Radiation source: fine-focus sealed tube	*R*_int_ = 0.033
graphite	θ_max_ = 27.5°, θ_min_ = 3.3°
ω scans	*h* = −14→14
5664 measured reflections	*k* = −10→10
1934 independent reflections	*l* = −14→14

### Refinement

**Table:**

Refinement on *F*^2^	Primary atom site location: structure-invariant direct methods
Least-squares matrix: full	Secondary atom site location: difference Fourier map
*R*[*F*^2^ > 2σ(*F*^2^)] = 0.041	Hydrogen site location: inferred from neighbouring sites
*wR*(*F*^2^) = 0.101	H atoms treated by a mixture of independent and constrained refinement
*S* = 1.03	*w* = 1/[σ^2^(*F*_o_^2^) + (0.0421*P*)^2^ + 0.247*P*] where *P* = (*F*_o_^2^ + 2*F*_c_^2^)/3
1934 reflections	(Δ/σ)_max_ = 0.001
122 parameters	Δρ_max_ = 0.23 e Å^−^^3^
1 restraint	Δρ_min_ = −0.23 e Å^−^^3^

### Fractional atomic coordinates and isotropic or equivalent isotropic displacement parameters (Å^2^)

**Table:**

	*x*	*y*	*z*	*U*_iso_*/*U*_eq_	
N1	0.36080 (11)	0.49391 (15)	0.56127 (12)	0.0188 (3)	
H1	0.4485 (9)	0.479 (2)	0.6153 (13)	0.024 (4)*	
N2	0.36741 (11)	0.89784 (15)	0.72193 (11)	0.0205 (3)	
N3	0.18404 (11)	0.69151 (16)	0.51024 (12)	0.0221 (3)	
C1	0.29407 (14)	0.36077 (18)	0.46992 (13)	0.0181 (3)	
C2	0.37632 (14)	0.23785 (18)	0.45198 (14)	0.0201 (3)	
H2	0.4717	0.2479	0.5007	0.024*	
C3	0.32046 (15)	0.1019 (2)	0.36410 (15)	0.0246 (3)	
H3	0.3775	0.0188	0.3534	0.029*	
C4	0.18124 (15)	0.0862 (2)	0.29138 (15)	0.0255 (3)	
H4	0.1427	−0.0058	0.2293	0.031*	
C5	0.09930 (14)	0.20604 (19)	0.31037 (14)	0.0235 (3)	
H5	0.0040	0.1948	0.2617	0.028*	
C6	0.15425 (14)	0.34292 (18)	0.39962 (14)	0.0204 (3)	
H6	0.0969	0.4236	0.4125	0.025*	
C7	0.31216 (13)	0.64466 (17)	0.58462 (13)	0.0174 (3)	
C8	0.40342 (13)	0.74982 (17)	0.69058 (14)	0.0184 (3)	
H8	0.4941	0.7127	0.7412	0.022*	
C9	0.23812 (14)	0.94629 (19)	0.64641 (14)	0.0232 (3)	
H9	0.2078	1.0522	0.6649	0.028*	
C10	0.14961 (14)	0.84350 (19)	0.54274 (15)	0.0245 (3)	
H10	0.0594	0.8820	0.4914	0.029*	

### Atomic displacement parameters (Å^2^)

**Table:**

	*U*^11^	*U*^22^	*U*^33^	*U*^12^	*U*^13^	*U*^23^
N1	0.0140 (6)	0.0182 (6)	0.0186 (6)	0.0013 (5)	0.0023 (5)	−0.0019 (5)
N2	0.0201 (6)	0.0197 (6)	0.0210 (6)	−0.0006 (5)	0.0085 (5)	−0.0008 (5)
N3	0.0182 (6)	0.0214 (7)	0.0220 (6)	0.0020 (5)	0.0048 (5)	−0.0008 (5)
C1	0.0201 (7)	0.0171 (7)	0.0147 (6)	−0.0012 (5)	0.0057 (5)	0.0009 (5)
C2	0.0176 (7)	0.0223 (8)	0.0194 (7)	−0.0005 (6)	0.0074 (6)	0.0000 (6)
C3	0.0285 (8)	0.0227 (8)	0.0257 (8)	−0.0008 (6)	0.0150 (6)	−0.0045 (6)
C4	0.0286 (8)	0.0236 (8)	0.0233 (7)	−0.0072 (6)	0.0108 (6)	−0.0075 (6)
C5	0.0197 (7)	0.0251 (8)	0.0214 (7)	−0.0047 (6)	0.0051 (6)	−0.0003 (6)
C6	0.0189 (7)	0.0197 (7)	0.0194 (7)	−0.0005 (6)	0.0056 (6)	0.0008 (6)
C7	0.0180 (7)	0.0175 (7)	0.0164 (7)	−0.0003 (5)	0.0073 (5)	0.0019 (5)
C8	0.0155 (6)	0.0193 (7)	0.0187 (7)	0.0006 (5)	0.0060 (5)	0.0019 (5)
C9	0.0215 (7)	0.0211 (7)	0.0250 (7)	0.0039 (6)	0.0087 (6)	−0.0015 (6)
C10	0.0191 (7)	0.0237 (8)	0.0266 (8)	0.0058 (6)	0.0065 (6)	−0.0001 (6)

### Geometric parameters (Å, °)

**Table:**

N1—C7	1.3689 (17)	C3—H3	0.9500
N1—C1	1.4039 (17)	C4—C5	1.384 (2)
N1—H1	0.891 (9)	C4—H4	0.9500
N2—C8	1.3207 (18)	C5—C6	1.393 (2)
N2—C9	1.3488 (17)	C5—H5	0.9500
N3—C7	1.3335 (17)	C6—H6	0.9500
N3—C10	1.3458 (19)	C7—C8	1.4120 (19)
C1—C6	1.3944 (18)	C8—H8	0.9500
C1—C2	1.3978 (19)	C9—C10	1.378 (2)
C2—C3	1.381 (2)	C9—H9	0.9500
C2—H2	0.9500	C10—H10	0.9500
C3—C4	1.389 (2)		
			
C7—N1—C1	130.38 (12)	C4—C5—H5	119.5
C7—N1—H1	113.3 (10)	C6—C5—H5	119.5
C1—N1—H1	116.3 (10)	C5—C6—C1	119.56 (13)
C8—N2—C9	116.75 (12)	C5—C6—H6	120.2
C7—N3—C10	115.67 (12)	C1—C6—H6	120.2
C6—C1—C2	119.09 (13)	N3—C7—N1	121.64 (12)
C6—C1—N1	124.65 (13)	N3—C7—C8	121.03 (12)
C2—C1—N1	116.25 (12)	N1—C7—C8	117.32 (12)
C3—C2—C1	120.72 (13)	N2—C8—C7	122.44 (12)
C3—C2—H2	119.6	N2—C8—H8	118.8
C1—C2—H2	119.6	C7—C8—H8	118.8
C2—C3—C4	120.26 (14)	N2—C9—C10	120.58 (13)
C2—C3—H3	119.9	N2—C9—H9	119.7
C4—C3—H3	119.9	C10—C9—H9	119.7
C5—C4—C3	119.26 (14)	N3—C10—C9	123.53 (13)
C5—C4—H4	120.4	N3—C10—H10	118.2
C3—C4—H4	120.4	C9—C10—H10	118.2
C4—C5—C6	121.08 (13)		
			
C7—N1—C1—C6	−12.7 (2)	C10—N3—C7—N1	−179.30 (12)
C7—N1—C1—C2	168.41 (13)	C10—N3—C7—C8	0.36 (19)
C6—C1—C2—C3	1.1 (2)	C1—N1—C7—N3	−4.2 (2)
N1—C1—C2—C3	−179.89 (12)	C1—N1—C7—C8	176.09 (13)
C1—C2—C3—C4	0.5 (2)	C9—N2—C8—C7	−0.73 (19)
C2—C3—C4—C5	−1.5 (2)	N3—C7—C8—N2	0.3 (2)
C3—C4—C5—C6	0.9 (2)	N1—C7—C8—N2	−179.99 (12)
C4—C5—C6—C1	0.7 (2)	C8—N2—C9—C10	0.4 (2)
C2—C1—C6—C5	−1.7 (2)	C7—N3—C10—C9	−0.7 (2)
N1—C1—C6—C5	179.40 (13)	N2—C9—C10—N3	0.3 (2)

### Hydrogen-bond geometry (Å, °)

**Table:**

*D*—H···*A*	*D*—H	H···*A*	*D*···*A*	*D*—H···*A*
N1—H1···N2^i^	0.89 (1)	2.12 (1)	2.977 (2)	162 (1)

Symmetry codes: (i) −*x*+1, *y*−1/2, −*z*+3/2.
